# Defective titanium dioxide single crystals exposed by high-energy {001} facets for efficient oxygen reduction

**DOI:** 10.1038/ncomms9696

**Published:** 2015-10-23

**Authors:** Dan-Ni Pei, Li Gong, Ai-Yong Zhang, Xing Zhang, Jie-Jie Chen, Yang Mu, Han-Qing Yu

**Affiliations:** 1 CAS Key Laboratory of Urban Pollutants Conversion, Department of Chemistry, University of Science and Technology of China, Hefei 230026, China; 2 Department of Municipal Engineering, Hefei University of Technology, Hefei 230009, China

## Abstract

The cathodic material plays an essential role in oxygen reduction reaction for energy conversion and storage systems. Titanium dioxide, as a semiconductor material, is usually not recognized as an efficient oxygen reduction electrocatalyst owning to its low conductivity and poor reactivity. Here we demonstrate that nano-structured titanium dioxide, self-doped by oxygen vacancies and selectively exposed with the high-energy {001} facets, exhibits a surprisingly competitive oxygen reduction activity, excellent durability and superior tolerance to methanol. Combining the electrochemical tests with density-functional calculations, we elucidate the defect-centred oxygen reduction reaction mechanism for the superiority of the reductive {001}-TiO_2−*x*_ nanocrystals. Our findings may provide an opportunity to develop a simple, efficient, cost-effective and promising catalyst for oxygen reduction reaction in energy conversion and storage technologies.

## Abstract (web summary)

Titanium dioxide is not generally considered to be an effective oxygen reduction catalyst. Here, the authors show that nanostructured titanium dioxide, self-doped with oxygen vacancies and with exposed high-energy {001} facets, exhibits competitive oxygen reduction catalytic activity and durability.

## Graphical abstract

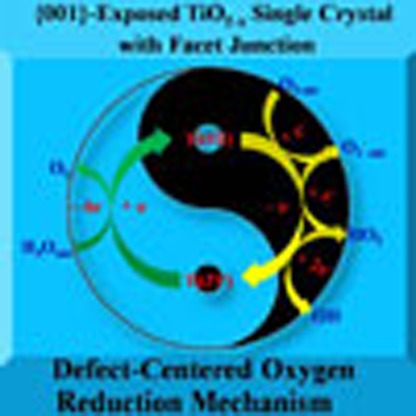

Increasing energy demands have stimulated intense research on alternative energy conversion and storage systems with a high efficiency, low cost and environmental benignity. The oxygen reduction reaction (ORR) has a key role in metal-air batteries and polymer membrane electrolyte fuel cells^1^,^2^, but has become a bottleneck. ORRs traditionally require the exclusive use of platinum-based catalysts^3^,^4^. Owing to the high cost of Pt and declining activity, alternative catalysts based on non-precious metal oxides, for example, Fe_3_O_4_, Co_3_O_4_ and so on, have been actively pursued^5^,^6^,^7^,^8^,^9^,^10^,^11^. TiO_2_ is a widely used semiconductor because of its Earth abundance, low cost, no toxicity and high stability^12^. Thus, incorporating TiO_2_ into energy conversion and storage systems is thus an attractive and promising idea. However, as a transition metal oxide, TiO_2_ has not been considered as an efficient electrocatalyst because of its low conductivity and poor reactivity resulting from intrinsic insulating properties, for example, the wide bandgap energy (*E*_g_=3.2 eV) (ref. 12). Up to now, several works have been reported on the use of TiO_2_ for ORR, but an overall low activity is obtained^12^,^13^,^14^,^15^,^16^,^17^,^18^,^19^.

Tremendous efforts have been made to improve TiO_2_ electrochemical properties. The non-stoichiometric reduction of TiO_2_ is known to substantially narrow the bandgap to the semiconductor level (usually below 2.0 eV) and thus increases the donor density and electric conductivities as well as the overall catalytic performance, because of the incorporated structural defects, that is, oxygen vacancy and Ti^3+^ (refs 20, 21, 22, 23, 24). On the other hand, it is well documented that the ORR at MnO_2_ proceeds through chemical oxidation of the surface Mn^3+^ generated by the discharge of MnO_2_ with O_2_ splitting, and thus the catalytic activity is dependent highly on the electrochemical redox activity of these species^25^,^26^,^27^,^28^,^29^. Mn^3+^ is the active valence state for ORR, owning to its single *e*_g_ occupancy and thus the enhanced ability to stabilize ORR intermediates on the catalyst surface^30^,^31^,^32^,^33^. For other Mn-based oxides, the high ORR activities are also proposed to be a result of surface non-stoichiometry and the presence of surface Mn^3+^ (ref. 29). In particular, Mn_3_O_4_ with a unique mixed-valence state has been used as an active catalyst, since the coexistence of Mn^2+^, Mn^3+^ and Mn^4+^ facilitates the formation of defects, that is, vacancies, electrons and holes, which govern the electronic distribution of Mn_3_O_4_ (refs 25, 26, 27, 28, 29, 30, 31, 32, 33).

The surface atomic structure, physiochemical properties and catalytic activity of TiO_2_ are also highly shape- and facet-dependent^34^. On the one hand, compared with randomly packed polycrystalline TiO_2_, the well-defined single crystals (SCs) with a high crystalline usually possess a much higher electronic conductivity and lower electron transfer resistance because of their continuous and ordered interior crystal structure, which favours the separation and transfer of reactive photocarriers as well as the overall catalytic performance^34^; on the other hand, compared with the thermodynamically stable {101} facet of TiO_2_ crystals with a low surface free energy (0.44 J m^−2^), the arrangement and category of constituent atoms on the high-energy {001} facet (0.90 J m^−2^) govern its unique geometrical and electronic structures, and its surface functional groups considerably affect its stability, adsorptive property and catalytic activity^34^. All these features make TiO_2_ crystals with {001} facet highly efficient for various catalytic applications.

Inspired by these informative results and the crystal similarity between TiO_2_ and MnO_2_, we try to explore the feasibility of using the reduced non-stoichimetric TiO_2−*x*_ SCs containing the electro-conducting Ti^3+^ species and oxygen vacancies as an ORR catalyst in energy conversion and storage systems. Herein, we first prepare the defective facet-controlled TiO_2−*x*_ SCs self-doped by oxygen vacancy via a facile solvothermal process, followed by a high-temperature thermal treatment in the reductive H_2_ atmosphere; then systematically explore the TiO_2−*x*_ SCs as a catalyst for ORR by both electrochemical tests and density-functional calculations (DFTs); at last, the reasons for the observed superiority of TiO_2−*x*_ SCs in ORR are investigated. The facet- and defect-engineered TiO_2−*x*_ SCs exhibit a surprisingly high ORR activity, excellent durability and superior tolerance to methanol, owing to their defect-centred oxygen reduction mechanism.

## Results

### Morphological and structural properties of TiO_2−*x*_ SCs

**Figure 1:**
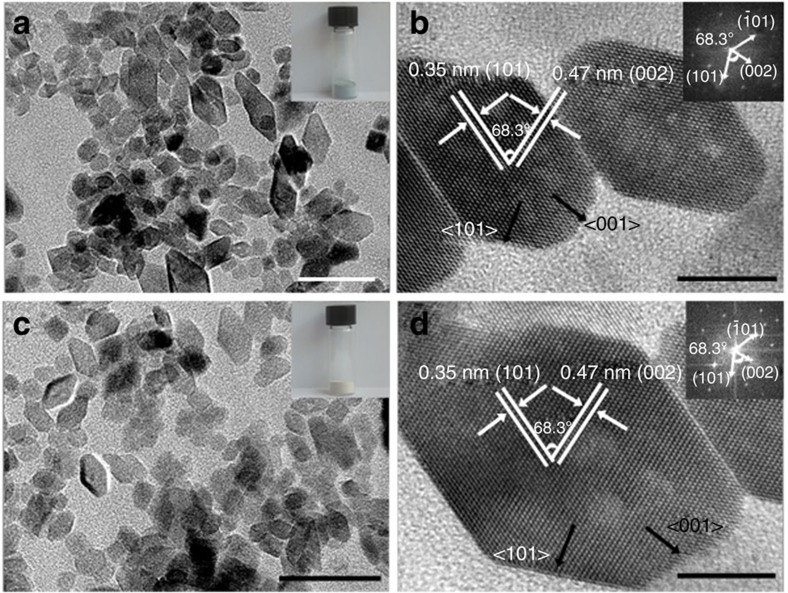
Morphology and structure of different TiO_2_ catalysts. Morphological (**a**,**c**) and structural (**b**,**d**) properties of the {001}-TiO_2−*x*_ SCs and {001}-TiO_2_ SCs, the insets of **b** and **d** are their corresponding fast-Fourier transform patterns. Scale bars, 50 nm (**a**,**c**) and 10 nm (**b**,**d**).

The morphology of the two prepared TiO_2_ samples were highly truncated bipyramids of about 20 nm in diameter, with few one-dimensional nanorods randomly packed (Fig. 1a,b and Supplementary Fig. 1). The lattice fringe spacing of 0.35 nm corresponded to the {101} planes, whereas the lattice fringe spacing of 0.47 nm corresponded to the {002} planes for the defective {001}-TiO_2−*x*_ SCs (Fig. 1b), indicating that the top/bottom surface exposed by truncation was bound by a {001} facet. Moreover, the angle labelled in the corresponding fast-Fourier transform image inset was 68.3°, which is in good agreement with the theoretical value of the angle between the {101} and {001} planes. The clear lattice fringes for the non-defective {001}-TiO_2_ SCs with an interplanar lattice spacing of 0.35 and 0.47 nm corresponded to the (101) and (002) atomic planes, respectively (Fig. 1d), and the angle labelled in the fast-Fourier transform image was also 68.3°. These results suggest that the morphology, structure and facet exposing of the defective {001}-TiO_2−*x*_ SCs were well retained after high-temperature calcination under oxygen-rich atmosphere (Fig. 1).

In the given F-free nonhydrolytic alcoholysis route, {001}-TiO_2−*x*_ SCs were formed via the controlled and smooth hydrolysis of TiCl_4_ in absolute ethanol^35^. Upon heating, ethanol was assumed to thermally react with dissolved oxygen and release water molecule (reaction 1), which coordinated to Ti^4+^ cations to form titanium oxy/hydroxychloride complex from polymerization/condensation. Then, TiO_2_ was formed with these complexes as intermediates (reaction 2). In this process, Cl^−^ anions might act as the functional capping and shape controlling reagent to favourably reduce the surface energy difference between the {001} and {101} facets^34^.


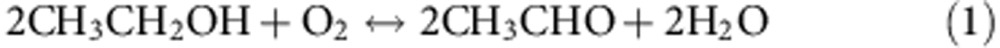



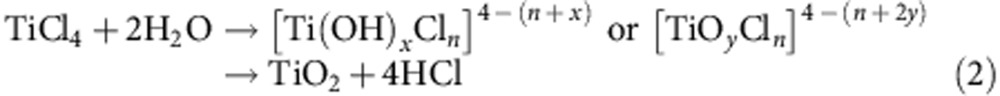


Supplementary Figure 2a shows the X-ray diffraction (XRD) patterns of the TiO_2_ polycrystals (PCs), TiO_2_ SCs and TiO_2−*x*_ SCs, in which the diffraction peaks highly matched those of anatase TiO_2_ (JCPDS No. 21-1272), whereas the broadening of the diffraction peaks of the two facet-engineered samples could be attributed to their relatively smaller crystal size^34^. On the other hand, the reduced TiO_2−*x*_ sample displayed type IV nitrogen adsorption-desorption isotherms with hysteresis loops indicative of the mesoporous structure with narrow pore size distribution centred around 15.0 nm (Supplementary Fig. 2b). These mesopores were constructed by gathering nanoparticles into nanosheets, and the Brunauer–Emmett–Teller (BET) surface area was calculated as 47.0 m^2^ g^−1^ from the nitrogen adsorption-desorption isotherms.

Compared with the conventional F-mediated hydrothermal/solvothermal routes^34^, the prepared TiO_2_ SCs possessed a smaller particle size and thus a higher surface area. These might provide more reactive sites for their catalytic applications, whereas the exposed percentage of high-energy {001} was relatively lower. Furthermore, the TiO_2−*x*_ SCs prepared using the Zn-mediated alcohothermal route in this work were very stable, and no obvious colour change was observed during its exposure in air even for over 1 year after their preparation. Their stability was comparable with the reduced TiO_2−*x*_ samples prepared by other methods^20^,^21^,^23^. The metallic Zn not only served as a functional reductant for the reductive formation of Ti^3+^, but also further stabilized the newly generated oxygen vacancies and hence Ti^3+^ on the crystal surface and subsurface. It also increased the number of surface oxygen vacancies by weakening the surface-oxygen bonds and thus enhanced the overall catalytic activity of defective TiO_2−*x*_ polymorph^35^.

### ORR activity of TiO_2−*x*_ SC

**Figure 2:**
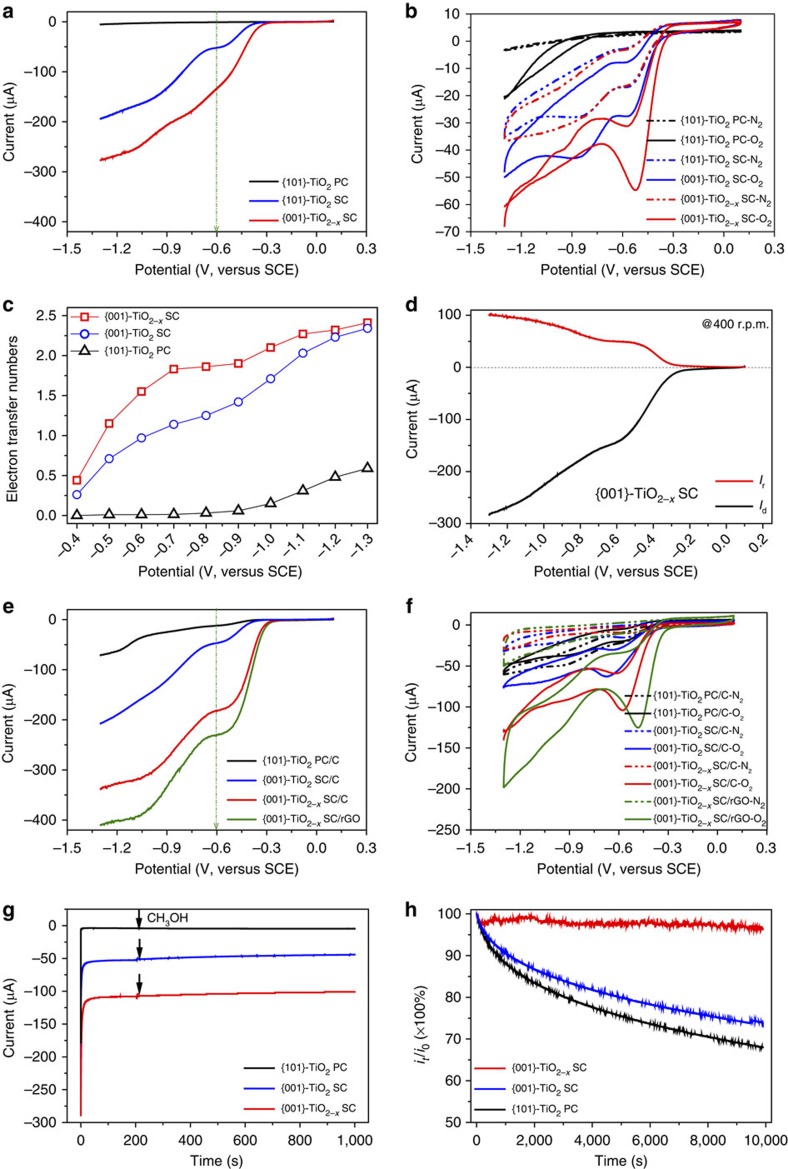
ORR properties on different TiO_2_ catalysts. Linear sweep voltammetry in O_2_-saturated 0.1 M aqueous KOH electrolyte solution at a scan rate of 10 mV s^−1^ at 400 r.p.m. (**a**,**e**) and cyclic voltammetry in N_2_- and O_2_-saturated 0.1 M KOH at a scan rate of 10 mV s^−1^ at 400 r.p.m. (**b**,**f**) electron transfer numbers calculated from the K-L model as a function of electrode potential (**c**) RRDE test on the defective TiO_2−*x*_ in O_2_-saturated 0.1 M aqueous KOH electrolyte solution at a scan rate of 10 mV s^−1^ at 400 r.p.m. (**d**) chronoamperometric responses obtained at −0.45 V in O_2_-saturated 0.1 M KOH at 200 r.p.m. with the addition of 10 vol.% methanol (**g**) and cathodic current stability obtained at −0.45 V in O_2_-saturated 0.1 M KOH at 200 r.p.m. (**h**).

Figure 2 shows that the ORR curves on both the rotating disk electrode (RDE) and the rotating ring-disk electrode (RRDE) were in the order of {001}-TiO_2−*x*_ SCs > {001}-TiO_2_ SCs > {101}-TiO_2_ PCs. The onset potential on the {101}-TiO_2_ PCs was beyond −1.0 V, whereas it positively shifted to approximately −0.4 V on the {001}-TiO_2_ SCs and further to −0.3 V on the {001}-TiO_2−*x*_ SCs (Fig. 2a), when the crystals were self-doped by oxygen vacancies and Ti^3+^ to form the reduced crystal structure. The cathodic current was also much higher on the facet- and defect-engineered TiO_2_ sample (Supplementary Table 1). At the given potential of −0.40 V (versus saturated calomel electrode (SCE)), the measured current density increased from 1.88 μA cm^−2^ on the {101}-TiO_2_ PCs to 39.55 μA cm^−2^ on the {001}-TiO_2_ SCs and further to 180.06 μA cm^−2^ on the {001}-TiO_2−*x*_ SCs (Fig. 2a).

The BET surface areas of the three involved TiO_2_ samples exhibited no significant difference (47.0, 43.2 and 42.3 m^2^ g^−1^ for {001}-TiO_2−x_ SCs, {001}-TiO_2_ SCs and {101}-TiO_2−*x*_ PCs, respectively; Supplementary Figs 2–4), and their gathering modes were identical, implying that these TiO_2_ samples have comparable electrochemical activities. Thus, the observed significant ORR superiority of the {001}-TiO_2−*x*_ SCs over the {001}-TiO_2_ and {101}-TiO_2_ polymorphs should be attributed mainly to the surface defect and {001} exposure (Fig. 2), rather than other sources. Also, the identical chemical compositions, crystal morphologies and structures of the {001}-TiO_2−*x*_ SCs and {001}-TiO_2_ SCs (Fig. 1 and Supplementary Figs 2,4 and 5) rendered them comparable electrochemical ORR activities. This indicates that the observed ORR activity difference between the two TiO_2_ samples dominantly originated from the defective crystal defects, oxygen vacancies and reduced Ti^3+^, rather than other sources. Moreover, the presence of coordination-unsaturated titanium sites increased the carrier density and facilitated the molecular diffusion of oxygen along the conducting crystal channels of the non-stoichiometric TiO_2−*x*_ SCs. The added Zn, served as both a reductant and a stabilizer, existed mainly in the form of ZnO clusters and dispersed on the TiO_2_ surface owning to the larger ionic radii of Zn^2+^ (0.88 Å) than Ti^4+^ (0.745 Å) (ref. 35). However, the incorporation of such a small amount of foreign ZnO as impurity, that is, ∼0.80 atom % in this work (Supplementary Fig. 5), would not bring about any ORR interference because of its low electrochemical activity as a wide bandgap n-type semiconductor^36^,^37^.

The number of transferred electrons in ORR, *n*, was calculated from the slopes of straight lines at different potentials using the Koutecky–Levich model (Supplementary Figs 6 and 7), and a continuous increase in *n* with the increasing overpotential was observed. Specifically, the calculated electron transfer number, *n*, was 0.44 at −0.4 V versus SCE and 2.41 at −1.3 V versus SCE for the defective {001}-TiO_2−*x*_ SCs (Fig. 2c), whereas only 0.26–2.34 and 0.00–0.59 for the perfect {001}-TiO_2_ SCs and the commercial {101}-TiO_2_ PCs, respectively. These calculation results indicate that the cathodic ORR on the three TiO_2_ polymorphs under the given conditions, from −0.4 V to −1.3 V versus SCE, was dominantly via the two-electron pathway with a high overpotential.

Such a two-electron pathway was further validated by the RRDE results (Fig. 2d) and is highly consistent with the DFT calculations. In the RRDE tests, the electrode modified with the defective {001}-TiO_2−*x*_ SCs generated a notable ring current, *I*_r_, over the whole potential range from the onset potential in 0.1 M KOH aqueous solution. This indicates the favourable and dominant generation of HO_2_^−^ intermediate in the electrochemical reduction. Specifically, for the defective {001}-TiO_2−*x*_ SCs, the measured H_2_O_2_ yield (HO_2_^−^% in alkaline solution) was 111.06% at the potential of −0.4 V versus SCE, and became 94.20% at −1.3 V versus SCE (Supplementary Fig. 8). The calculated electron transfer number, *n*, from the RRDE tests varied in a range of 1.78–2.17 with the potential of −0.4 to −1.3 V versus SCE (Supplementary Fig. 8). These RRDE results are consistent with the RDE results, suggesting that the ORR on the defective {001}-TiO_2−*x*_ SCs proceeded mainly via a two-electron pathway.

Considering the low electric conductivity and poor dispersity of nano-sized metal oxide catalysts, some nano-structured carbonaceous supports are usually needed in ORR tests^18^. Herein, all the TiO_2_ samples were mixed with the commercial Cabot carbon nanoparticles (Vulcan XC-72R, USA) as a conductive support. The same trend of ORR activity, {001}-TiO_2−*x*_ SCs/C>{001}-TiO_2_ SCs/C > {101}-TiO_2_ PCs/C, was also observed (Supplementary Table 1). The {001}-TiO_2−*x*_ SCs was hydrothermally deposited on reduced graphene oxide (rGO) to form the {001}-TiO_2−*x*_ SCs/rGO hybrid, and it exhibited a further enhanced capacity for ORR (Fig. 2e,f). Interactions between rGO and metal oxide indicate a possible charge transfer across the interface depending on the hybrid interface spacing and the Fermi level difference, which might be the main reason for the enhanced ORR activity^10^,^11^. In addition, all linear sweep voltammetry (LSV) curves exhibited a slow current increase and no current plateau (Supplementary Figs 6 and 9), indicating the two-electron reduction of O_2_ to OOH^−^. All these results clearly indicate that both the exposed high-index {001} facet and the engineered oxygen vacancy contributed to the enhanced ORR activity of the TiO_2_ catalyst.

The long-time ORR stability was evaluated using chronoamperometric response at −0.45 V in O_2_-saturated 0.1 M KOH aqueous solution (Fig. 2g,h). The defective TiO_2−*x*_ SCs exhibited excellent stability, retaining more than 95% of the initial current even after 10,000 s, whereas both the TiO_2_ SCs and TiO_2_ PCs lost nearly 30% of the initial current. Moreover, all TiO_2_ samples showed superior selectivity to ORR with no obvious current change after adding methanol. In addition, the TiO_2−*x*_ SCs also survived in the highly corrosive electrolyte (6 M KOH aqueous solution), demonstrating its high activity, favourable kinetics and excellent stability. The observed superior ORR stability of the defective TiO_2−*x*_ SCs should be attributed mainly to the excellent stability of the surface-engineered Ti^3+^ species, because the metallic Zn not only served as a chemical reductant for the reductive formation of Ti^3+^ but also further stabilized the newly generated oxygen vacancies and Ti^3+^ on the crystal surface and subsurface^35^. The poor ORR catalytic stability of the TiO_2_ PCs was probably owning to their anatase-rutile mixed crystal phase^38^,^39^.

The above results clearly show that the ORR activity of TiO_2_ was substantially enhanced by a simple facet- and defect-engineering strategy. It is noted that the prepared defect-engineered TiO_2−*x*_ with exposed high-energy {001} facets still exhibited a lower ORR activity than Fe_3_O_4_ and Mn_3_O_4_ (refs 10, 32), the two most typical metal oxide ORR electrocatalysts, with a more negative onset potential of ca −0.30 V and a lower cathodic current density of ca 1.0 mA cm^−2^ (Fig. 2a,b). On the other hand, its ORR stability was much higher than the commercial Pt/C benchmark, as the chronoamperometric response remained intrinsically unchanged on the TiO_2−*x*_ while more than 20% decrease was observed on the Pt/C benchmark during the given 10,000 s (refs 4, 5, 9). Moreover, TiO_2_ is intrinsically characterized of Earth abundance, low cost, high stability and environmental compatibility for industrial applications.

### Ti^3+^ and its roles on TiO_2−*x*_ SCs

**Figure 3:**
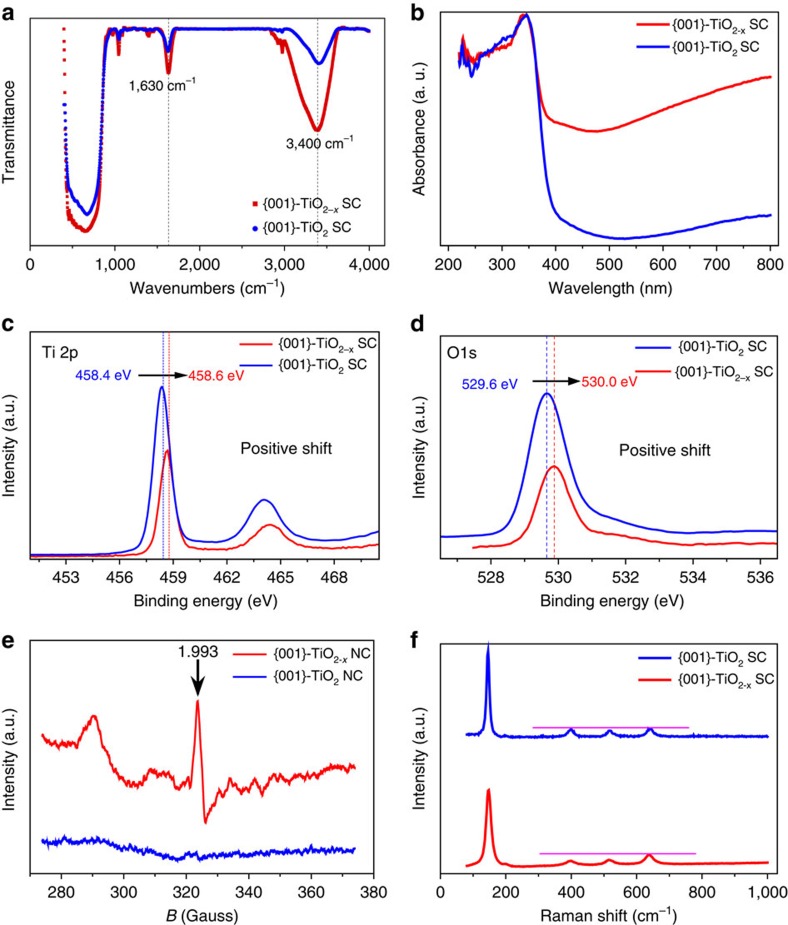
Defects in TiO_2−*x*_ SCs. Determination of defective Ti^3+^ in the {001}-engineered TiO_2−*x*_ SCs: FTIR (**a**), diffuse reflectance spectra (**b**), XPS (**c**,**d**), ESR (**e**) and Raman (**f**).

The surface Ti oxidation state was probed by Fourier transform infrared (FTIR) spectroscopy, diffuse reflectance spectra, X-ray photoelectron spectroscopy (XPS), electron spin resonance (ESR) and Raman spectra, respectively (Fig. 3). The FTIR spectrum exhibited two distinct -OH stretching bands at 3,400 and 1,632 cm^−1^ (Fig. 3a), indicating the presence of oxygen vacancies and/or reductive Ti^3+^ as the reactive adsorption sites for water molecules and hydroxyl ions. The wider -OH absorption bands also suggest that the -OH groups experienced a more varied environment on the defective {001}-TiO_2−*x*_ SCs. In high-resolution XPS, an obvious shoulder appeared in the low-energy range (457.5–455.5 eV) of the Ti 2p spectrum, which might be attributed to Ti^3+^ in the TiO_2_ lattice (Fig. 3c,d)^22^. This was further confirmed by the ESR spectrum with the strong signals around *g*=1.95 and *g*=2.00, respectively^22^, but no significant signal indicative of surface Ti^3+^ was observed at *g*=2.02 because of the surface oxidation (Fig. 3e). Moreover, the measured Raman spectra also confirmed the presence of more oxygen vacancies in the reduced TiO_2_ SCs, owing to the positive peak shift, which was resulted from the modified geometric and surface structures (Fig. 3f)^23^.

In principle, the Ti^3+^ species were generated from the reduction of Ti^4+^ under reductive conditions and the *in situ* Ti^3+^-doped TiO_2_ displayed oxygen vacancies because of the replacement of Ti^4+^ by Ti^3+^ in the lattice^20^,^21^,^22^,^23^,^24^. Meanwhile, the {001} facet contained more oxygen vacancies than the {101} facet, because each Ti in the {001} facet coordinated with 5 oxygen atoms, whereas each Ti in the {101} facet coordinated with either 5 or 6 oxygen atoms in the probability around 50% to 50% (ref. 34). The presence of Ti^3+^ and the oxygen vacancies resulted in some new energy levels below the conduction band of the TiO_2_ (refs 22, 23), leading to an obvious spectral response in visible range (Fig. 3b). Such a photon excitation in the infrared region indicates that the formed Ti^3+^ was dominantly derived from oxygen deficiency^24^. In addition, the oxygen-deficient TiO_2_ SCs had different Raman spectra from the oxygen-free analogue, but they shared nearly the same XRD patterns. This result indicates that most oxygen deficiency-related Ti^3+^ should mainly locate on the surface and subsurface of the crystals^21^.

**Figure 4:**
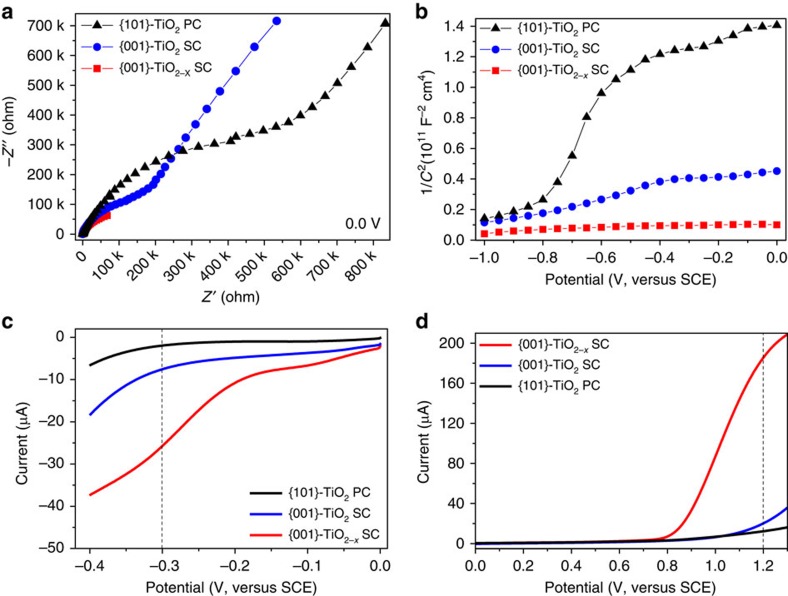
Ti^3+^-induced properties of TiO_2−*x*_ SCs. Role of defective Ti^3+^ in the electronic and electrochemical properties of the {001}-engineered TiO_2−*x*_ SCs: electrochemical impedance spectroscopy (**a**), Mott–Schottky (**b**), HER (**c**) and OER (**d**).

The presence of these structural defects played important roles in the electro-catalytic properties of the TiO_2−*x*_. The enhanced visible light adsorption on the reduced TiO_2_ SCs (Fig. 3b) indicates that the introduced new energy states by Ti^3+^ self-doping mainly laid below the bottom of conductance band, and these electro-conducting Ti^3+^ could significantly narrow the inherent bandgap and enhance the electrical conductivity (Fig. 4a and Supplementary Fig. 10). In addition, both TiO_2_ samples exhibited positive slopes as expected for n-type semiconductors, and a higher electron density was obtained for the TiO_2−*x*_ SCs from the slope of Mott–Schottky plot (Fig. 4b)^21^. Thus, although the TiO_2−*x*_ SCs were not the direct bandgap semiconductor (Fig. 3b), significant modifications of their electric structures occurred during the self-doping process, which contributes to their higher electron density. Moreover, all these enhanced electrochemical conductivity, reduced electron transfer resistance and improved interfacial capacity on the TiO_2−*x*_ SCs derived from the second semicircles in electrochemical impedance spectroscopy (associated with the fast reaction kinetics and considerable increases in the electrochemically accessible surface) are essential for an efficient ORR catalyst.

Lowering the electrochemical overpotentials for both hydrogen evolution reaction (HER) and oxygen evolution reaction (OER) favours their efficient applications. In this work, both much reduced overpotentials and substantially enhanced currents were obtained on the defective {001}-TiO_2−*x*_ SCs for both HER and OER (Fig. 4c,d). For example, the cathodic current at the selected −0.3 V (versus SCE) and the anodic current at the selected +1.2 V (versus SCE) were −1.9 μA and 12.3 μA on the {101}-TiO_2_ PCs, −7.5 μA and 20.2 μA on the {001}-TiO_2_ SCs and −25.7 μA and 185.6 μA on the {001}-TiO_2−*x*_ SCs, respectively. The catalytic activity order of {001}-TiO_2−*x*_ SCs >{001}-TiO_2_ SCs > {101}-TiO_2_ PCs indicates that the introduction of defective oxygen vacancies uniformly into TiO_2_ lattice and the exposed high-energy {001} facets could substantially improve their catalytic performance.

### Catalytic mechanism of TiO_2−*x*_ for ORR

**Figure 5:**
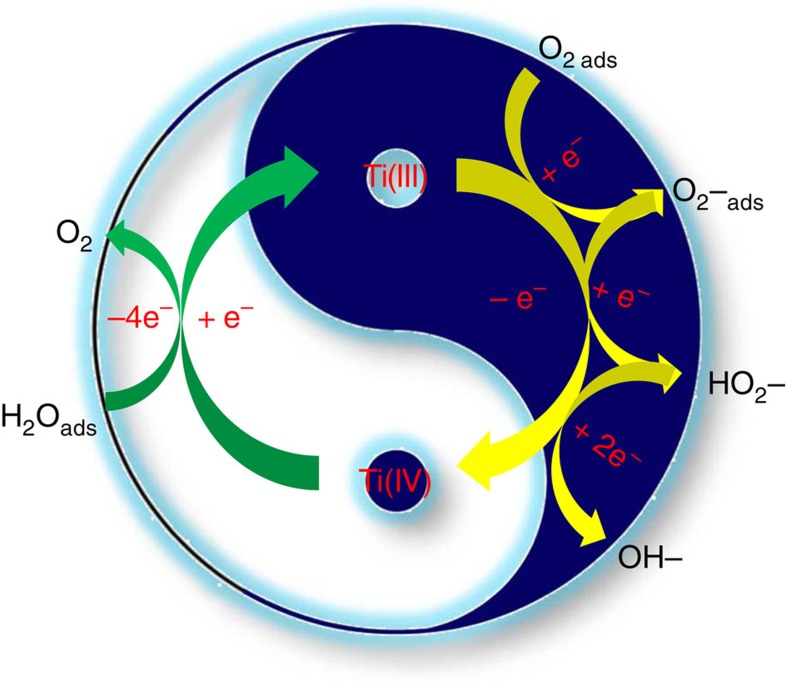
Defect-centred ORR mechanism on TiO_2−*x*_ SCs. The mixed-valence Ti species cycling between +3 and +4 states at oxygen vacancy sites in the defective TiO_2−*x*_ SCs thermodynamically served as the reactive sites for cathodic oxygen reduction.

The ORR is affected by the chemical composition, elemental valence, crystal structure and surface state^5^,^6^,^7^,^8^,^9^,^10^,^11^. Also, the adsorption configuration of surface oxygen molecule and the corresponding strength of oxygen binding also impact with the ORR activities. In this work, the enhanced ORR activity on the defective {001}-TiO_2−*x*_ SCs might be highly associated with the single-crystalline structure, the exposed high-energy {001} facet and the Ti multiple valences caused by the defective oxygen vacancies (Fig. 5). First, the significant enhancement of electric conductivity from both the continuous and ordered interior structure and the enhanced crystallinity should greatly favour fast electron transfer (that is, higher charge mobility) and reduce electrode polarization in ORR. Second, the exposed high-energy {001} facet with unique atomic, electronic and energetic structure could ensure its significant ORR activity because of its facet-mediated behaviours in dissociative adsorption and charge mobility^34^. Last, the active valence state of defective Ti^3+^ originating from engineered oxygen vacancies could play a governing role in ORR and thus the enhanced ability to stabilize intermediates on catalyst surface owning to its single *e*_g_ occupancy^40^.

In TiO_2_, the missed oxygen atom from the bulk or surface is taken by one or two ‘free' electrons in the defective crystal, and the three nearest Ti atoms tend to relax away from the vacancy in order to strengthen their bonding with the rest of the lattice^20^. Thus, the defect-related properties mainly include structural, electronic, optical, dissociative adsorption and reductive properties^21^. For ORR, the chemical adsorption and dissociative activation of O_2_ on the TiO_2_ surface is a governing step, yet it has been well documented that both of the two processes are thermodynamically favoured only on the defective negative site, rather than the perfect neutral site^22^,^23^. In principle, the dissociated adsorption of O_2_ on the defective oxygen vacancy could result in more efficient electron-transfer process and greater ORR kinetics because of the strong coupling between the adsorbate and the surface, and are intimately related to the overall catalytic performance.

On the defective {001}-TiO_2−*x*_ SC, O_2_ is first selectively adopted onto the defective subsurface oxygen vacancy with a much higher adsorption energy (reaction 3), as it only adsorbs onto the site where excess negative charge is available to form O-Ti bond; then, the chemically adsorbed O_2_ could capture the free electrons located on oxygen vacancy site, simultaneously producing superoxide radical groups (reaction 4). The formation of these radical groups is effective to promote charge separation as well as the reduction of O_2_ via either a two-electron pathway (reactions 5 and 6) or a 4-electron pathway (reactions 7 and 8), followed by the reduction of Ti^4+^ to Ti^3+^ (reaction 9). In this defect-centred ORR (Fig. 5), the possible catalytic disproportionation of hydrogen peroxide occurs (reactions 7 and 8), which might be the overall rate-limiting step of the ORR.


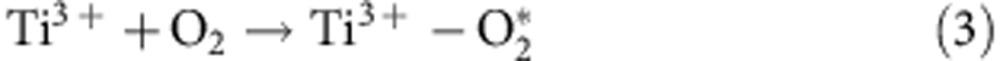



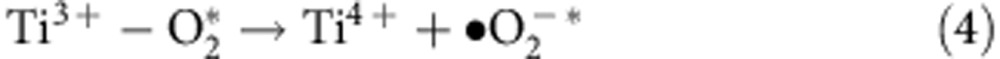



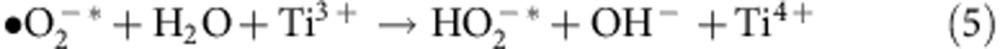



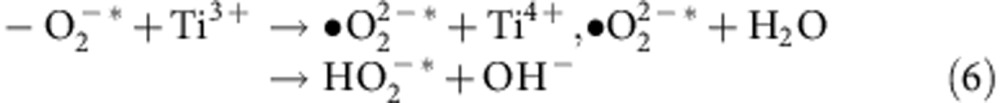



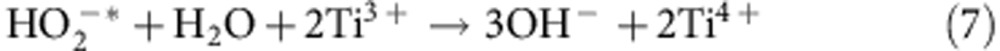



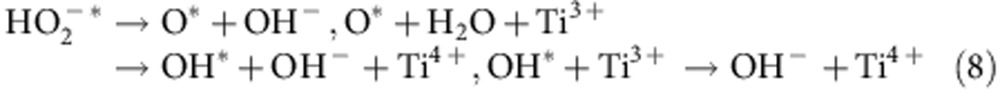



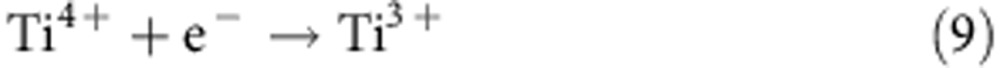


**Figure 6:**
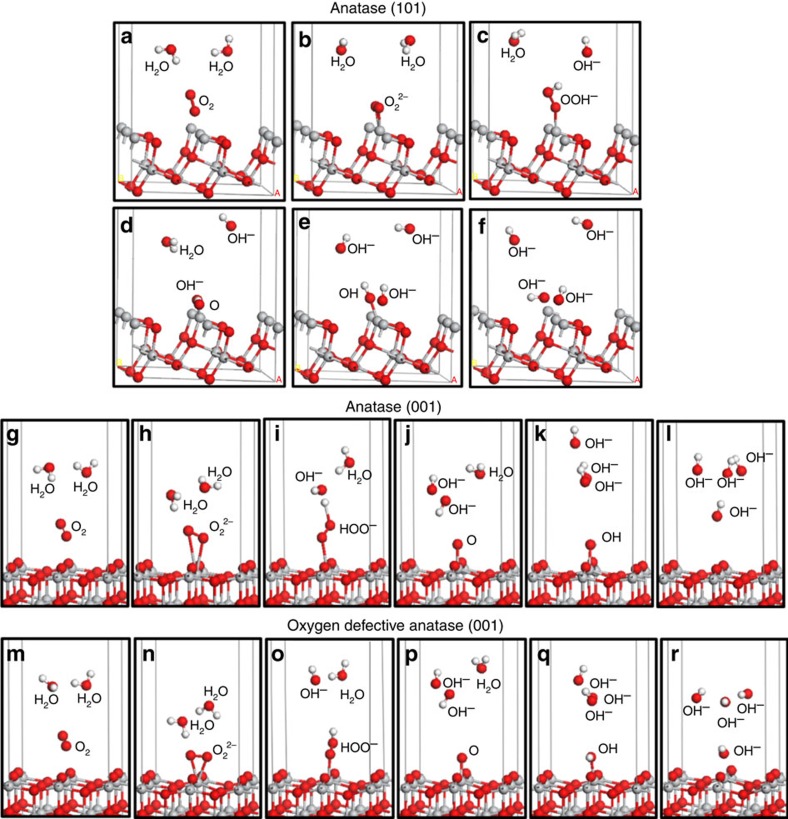
Geometry structures of adsorption configuration. Optimized structures of each step in ORR on the {101}-TiO_2_, {001}-TiO_2_ and {001}-TiO_2−*x*_. (**a,g,m**) Initial structure, (**b,h,n**) O_2_^2−^*, (**c,i,o**) HOO^−^*, (**d,j,p**) O*, (**e,k,q**) OH* and (**f,l,r**) OH^−^, white, red and grey spheres indicate H, O and Ti atoms, respectively.

**Figure 7:**
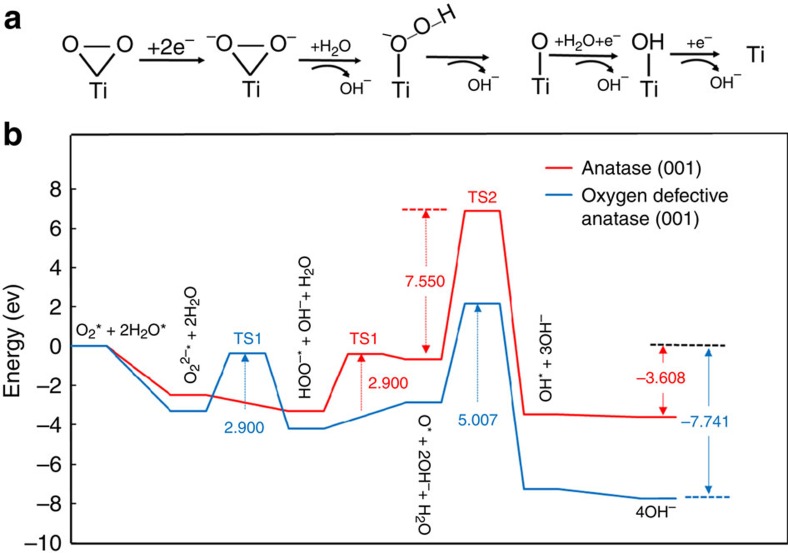
ORR mechanism and energy profiles. DFT-calculated ORR mechanism on the anatase TiO_2_ surface (**a**), and the energy profiles of ORR on the {001}-TiO_2_ and {001}-TiO_2−*x*_, TS represents transition states (**b**).

The DFT calculations further indicate that the four- and fivefold coordinated vacancy-surrounding titanium sites assume their respective role in oxygen adsorption and reactive activation, which lower the activation barrier and avoid catalytic poisoning. On the one hand, oxygen was found to be strongly bound onto the {001}-TiO_2−*x*_ with the highest adsorption energy of 2.28 eV (Table 1 and Supplementary Note 1); on the other hand, based on the optimized geometry structures of adsorption configuration (Fig. 6), the free energies (*G*) of key intermediates, O_2_^−^*, O_2_^2–^*, HOO^−^*, OH* and O* (*: adsorbed state; Supplementary Table 2 and Supplementary Note 1), and free energy changes (Δ*G*) of each step of ORR, in Fig. 7a, were further calculated (Supplementary Table 3) and the favourable energy profile is plotted on {001}-TiO_2−*x*_ (Fig. 7b). First, the Δ*G* of −3.297 eV for O_2_^2–^* formation is the lowest on {001}-TiO_2−*x*_, compared with that of 3.945 and −2.478 eV on {101}-TiO_2_ and {001}-TiO_2_; second, for the overall rate-limiting ORR step of regenerating hydroxide from adsorbed oxide, {001}-TiO_2−*x*_ affords the lowest energy barrier of only 5.01 eV, compared with that of 10.58 and 7.55 eV on {101}-TiO_2_ and {001}-TiO_2_; at last, the total Δ*G* from adsorbed O_2_ to 4OH^−^ on {001}-TiO_2_, {001}-TiO_2−*x*_ and {101}-TiO_2_ is −3.608 −7.741 and −2.806 eV, respectively. All these results not only confirm that {001}-TiO_2_ was thermodynamically and kinetically more active than {101}-TiO_2_, but also show that oxygen vacancies on {001}-TiO_2−*x*_ effectively increased its catalytic activities. This conclusion is consistent with the electrochemical results (Fig. 2 and Supplementary Figs 6 and 9).

Although the further reduction of the generated peroxide on the defective {001}-TiO_2−*x*_ SCs showed a high-energy barrier of 5.01 eV, its Gibb's free energy was −4.43 eV (Supplementary Table 3), indicating that this step could spontaneously occur at room temperatures despite of a low reaction rate. Thus, the ORR reaction equilibrium was able to gradually shift to the reaction direction of deep oxygen reduction. Moreover, this theoretical ORR rate-controlling step with such a high-energy barrier for the subsequent reduction of the as-generated peroxide from the DFT calculations was further validated by both the RDE and RRDE tests. The calculated electron transfer number and peroxide percentage were ∼2.2% and ∼95%, respectively, even at a highly negative −1.3 V versus SCE on the defective {001}-TiO_2−*x*_ SCs (Fig. 2c and Supplementary Fig. 8). Also, the geometric structural changes of this rate-controlling step were simple and lie in the last H_2_O dissociation and OH* generation. Thus, the series two-electron mechanism is the sole ORR pathway for this step.

## Discussion

This mixed-valence in the defective TiO_2−*x*_ as the most active catalyst for defect-centred ORR is also consistent with the previous design principle for perovskite catalysts, especially on Mn-based oxides^25^,^26^,^27^,^28^,^29^,^30^,^31^,^32^,^33^. In addition, as the ORR involves the oxidation and reduction of surface-defective Ti species, the number and activity of these redox centres could be important factors to define the overall catalytic performance.

In summary, we show that the new form of TiO_2_ polymorph with defective crystal structure can be exploited as a promising ORR catalyst with a competitive activity, excellent durability and sufficient selectivity. The exposed high-energy {001} facets of the TiO_2_ SCs is advantageous for oxygen interfacial adsorption and dissociative activation in ORR process. Moreover, the crystal oxygen vacancies and reductive Ti^3+^ sites further guarantee a durable and facile ORR process via a defect-centred mechanism. Our findings might open up a new avenue for efficient energy conversion and storage technologies based on the Earth-abundant, scalable, non-precious metal catalysts.

## Methods

### TiO_2−*x*_ SCs preparation

The reduced TiO_2−*x*_ SCs engineered by the high-index {001} facet were prepared using a modified solvothermal method^34^. Briefly, at room temperature (10–15 °C), 2-ml TiCl_4_ solution was slowly added into the absolute ethanol (40 ml) under vigorous stirring to form a transparent solution; then a given amount of metallic zinc powers with Ti/Zn molar ratio of 4:1 was added into the solution and magnetically stirred for over 1 h, in which process the solution colour gradually became blue; the obtained solution was immediately transferred into a 50-ml Teflon-lined stainless steel autoclave and was kept under 200 °C for 24 h; after this alcohothermal treatment and being cooled down to room temperature, the solution was subjected to high-speed centrifugation; the precipitate was collected and repeatedly washed with EtOH and water for several times, dried in vacuum at 60 °C and finally ground to obtain blue-coloured Ti^3+^ self-doped TiO_2−*x*_ for use. For comparison, the commercial TiO_2_ PCs with low-index {101} facet (P25, Degussa Co. , mean particle size of about 25 nm, anatase/rutile=80:20, BET surface area of 50 m^2^ g^−1^) and the oxidized TiO_2−*x*_ SCs calcinated at 600 °C for 2 h under oxygen-rich atmosphere were used for reference.

### Material characterization

The morphology and structure were characterized by high-resolution transmission electron microscopy (JEM-2100, JEOL Co.). XRD (X'Pert, PANalytical BV) was used to analyse the crystal structure. The diffuse reflectance spectra were measured on a ultraviolet–visible spectrophotometer (UV 2550, Shimadzu Co.). The chemical compositions were characterized using XPS (PHI 5600, Perkin-Elmer Inc.) and Raman spectrum (LABRAM-HR, JY Co.). The electronic state of Ti and O atoms were measured to provide structural information by ESR (JES-FA200, JEOL Co.). The surface area was measured using the BET method with a Builder 4200 instrument (Tristar II 3020M, Micromeritics Co.) at liquid nitrogen temperature. The infrared spectra were recorded between 4,000 and 400 cm^−1^ with a FTIR spectrometer (Magna-IR 750, Nicolet Instrument Co.) using a potassium bromide disc technique.

### Electrochemical characterization

All electrochemical measurements were carried out in a home-made three-electrode system, with pure and modified TiO_2_ SCs deposited on glass carbon as the working electrode, Pt wire as the counter electrode and Ag/AgCl (KCl, 3 M) as the reference electrode. Electrochemical impedance spectroscopy analysis was conducted by applying an AC voltage amplitude of 5 mV within the frequency range from 10^5^ to 10^−2^ Hz in 10 g l^−1^ NaCl aqueous solution; Mott–Schottky plots were measured in 0.1 M Na_2_SO_4_ aqueous solution by impedance measurement at the fixed frequency of 1,000 Hz between the applied voltage range of 0–1 V; HER and OER were carried out in 0.5 M H_2_SO_4_ aqueous solution and 0.1 M KOH aqueous solution, respectively, at a scan rate of 50 mV s^−1^.

### ORR tests

Electrochemical ORR, LSV and cyclic voltammetry measurements, on the glass carbon RDE (dimension of 5 mm, geometric surface area of 0.196 cm^2^; Pine Research Instrumentation Inc.), were performed using a computer-controlled potentiostat (CHI 760D, CH Instrument) with a three-electrode glass electrochemical cell. The TiO_2_ sample (2.0 mg) was dispersed in 2 ml absolute iso-propanol solvent. After applying ultrasonification for 30 min, 20 μl of the homogeneous suspension was dropped and the TiO_2_ nanoparticles were adhered to the RDE using Nafion solution (5 μl, 0.05 wt. %) with a catalyst loading of ∼0.10 mg cm^−1^. The ink was dried slowly in open air and the drying condition was adjusted by trial and error until a uniform catalyst distribution across the electrode surface was obtained^5^,^9^. The RDE loaded with different TiO_2_ samples was used as the working electrode, an SCE as the reference electrode, and a Pt wire, as the counter electrode. The electrochemical ORR tests were conducted in either N_2_- or O_2_-saturated electrolyte (with 0.1 M KOH) at room temperature (10–15 °C). After purging O_2_ or N_2_ in the electrolyte for 30 min, the potential range was cathodically scanned between −1.3 and +0.1 V versus SCE at a scan rate of 10 mV s^−1^. The LSV measurements were conducted at different rotating speeds from 400 to 1,600 r.p.m., using a Pine Model, whereas the cyclic voltammetry measurements were carried out without electrode rotating. In the ORR tests, the working electrode was cycled at least 15 times before electrochemical data were recorded, and a continuous flow of N_2_ or O_2_ was maintained to ensure saturation in supporting electrolyte. The ORR kinetic parameters are calculated using the Koutecky–Levich model:


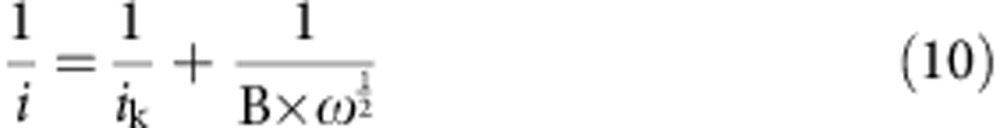


where *i* is the measured current, *i*_k_ is the kinetic current and *ω* is the electrode rotation rate.

The theoretical value of the Levich slope (*B*) is evaluated from the following equation:


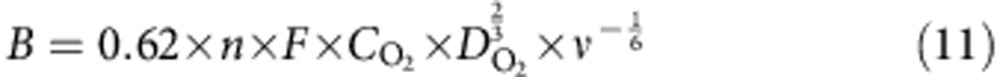


where *n* is the electron transfer number in ORR, *F* is the Faradic constant (96,485 C mol^−1^), *C*_O2_ is the saturated oxygen concentration in 0.1 M KOH aqueous solution (1.2 × 10^−6^ mol cm^−3^), *D*_O2_ is the oxygen diffusion coefficient (1.73 × 10^−5^ cm^2^ s^−1^) and *ν* is the kinematic viscosity of the solution (0.01 cm^2^ s^−1^).

Electrochemical ORR measurements on the glass carbon RRDE (disk electrode: dimension of 5 mm, geometric surface area of 0.196 cm^2^; Pine Research Instrumentation Inc.) were carried out by the same procedures as on the RDE. The disk electrode was scanned cathodically at a rate of 10 mV s^−1^ and the ring potential was kept constant at +0.3 V versus SCE. The HO_2_^−^ percentage and the electron transfer number, *n*, are determined by the following equations:


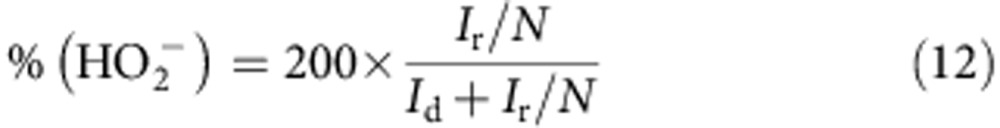



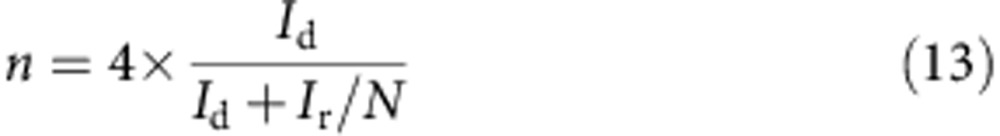


where *I*_d_ is disk current, *I*_r_ is ring current and *N* is current collection efficiency of the Pt ring, which was determined to be 0.40 from the reduction of K_3_Fe[CN]_6_.

### DFT calculations

DFT calculations were carried out using the ultrasoft pseudopotential for electronic–ion interactions and generalized gradient approximation with the Predew–Burke–Ernzerhof exchange for exchange-correlation functional in the supercell approach as implemented in CASTEP program^41^,^42^. The convergence criteria was set out to be *express*, and the tolerances of the energy was 1 × 10^−3^ eV per cell. The norm-conserving pseudopotential was constructed using the pseudopotential generator implemented in CASTEP. The energy cutoff of 750 eV for the plane wave basis was used throughout the study. The Brillouin zone integration was obtained with variable numbers of *k*-points, depending on the unit cell size and shape, generated by the Monkhorst-pack algorithm. Phonon dispersion and density of states were computed using the method of Finite displacement. The linear synchronous transit/quadratic synchronous transit methods were employed to search for the transition state structure and acquire the activation energy (*E*_a_) of ORR^43^.

The adsorption energy (Δ*E*_ads_) was calculated to estimate the strength of a molecule–surface interaction and to seize the most energetically stable adsorption model. The Δ*E*_ads_ could be expressed as:


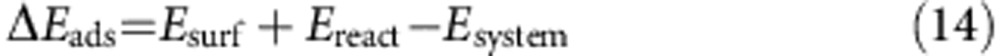


where the total energy values *E*_system_, *E*_surf_, *E*_react_ are relative to supercells simulating a molecule interacting with anatase TiO_2_ surfaces, the surfaces and an isolated molecule in the vacuum, respectively.

The Gibb's free energy difference (Δ*G*) of each elementary reaction was given by the following equation:


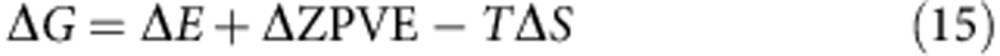


where Δ*E* is the total energy change, directly obtained from DFT calculations, ΔZPVE is the change in zero-point energies, *T* is temperature (298.15 K) and Δ*S* is the change in entropy.

For the surface models, a vacuum region of 15 Å was embedded along surface to avoid the unwanted interaction between the slab and its period images. The {001}-TiO_2_ surface had fivefold coordinated Ti atoms only, interconnected at the surface by two-coordinate bridging oxygen atoms, whereas the {101}-TiO_2_ surface exhibited a sawtooth-like corrugation consisting of 5-coordinate and 6-coordinate Ti atoms as well as 2-coordinate and 3-coordinate oxygen atoms. In addition, according to the experiments, the {001}-TiO_2−*x*_ surface was constructed from the perfect {001}-TiO_2_ surface model with a subsurface oxygen vacancy.

## Additional information

**How to cite this article:** Pei, D.-N. *et al*. Defective titanium dioxide single crystals exposed by high-energy {001} facets for efficient oxygen reduction. *Nat. Commun.* 6:8696 doi: 10.1038/ncomms9696 (2015).

## Supplementary Material

Supplementary Information Supplementary Figures 1-10, Supplementary Tables 1-3, Supplementary Note 1 and Supplementary References

## Figures and Tables

**Table 1:** Adsorption energy (Δ*E*_ads_) for the three different adsorption configurations of O_2_ on {001}-TiO_2_ and {001}-TiO_2−*x*_.

**System**	***E***_**surf**_ **(eV)**	***E***_**react**_ **(eV)**	***E***_**system**_ **(eV)**	Δ***E***_**ads**_ **(eV)**
{001}-TiO_2−*x*_	−20,794.63	−857.85	−21,654.76	2.28
{001}-TiO_2_	−21,226.38	−857.85	−22,085.26	1.03
