# Supplementary material for: Defective titanium dioxide single crystals exposed by high-energy {001} facets for efficient oxygen reduction
Source: Nat Commun. 2015 Oct 23;6:8696. doi: 10.1038/ncomms9696 (PMC4846326; doi:10.1038/ncomms9696)
Supplement: Supplementary Information — Supplementary Figures 1-10, Supplementary Tables 1-3, Supplementary Note 1 and Supplementary References [file ncomms9696-s1.pdf]

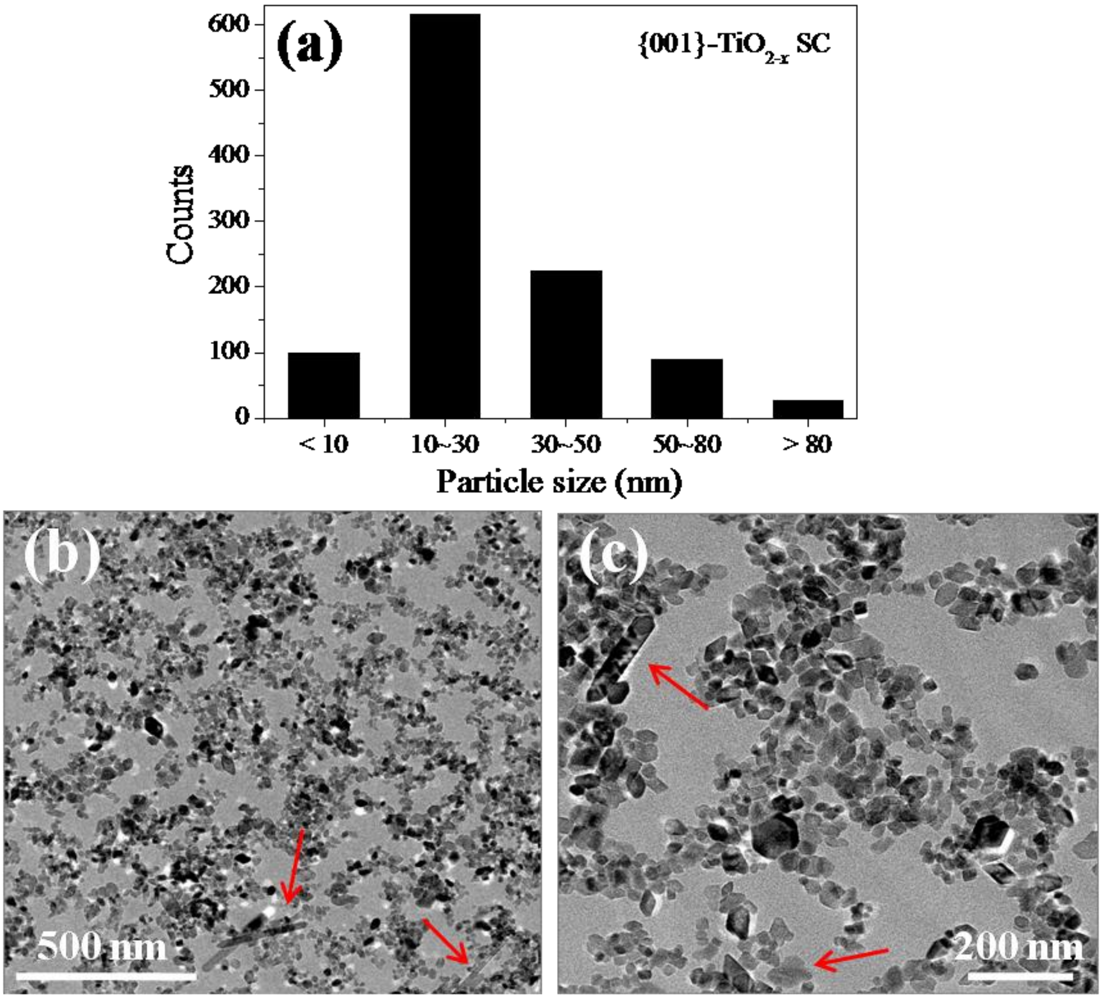

Supplementary Figure 1 | Morpholigical properties of TiO_{2-x} SCs. The statistical particle size distribution (**a**) of the defective {001}-TiO_{2-x} SCs and their typical TEM images (**b**, **c**).

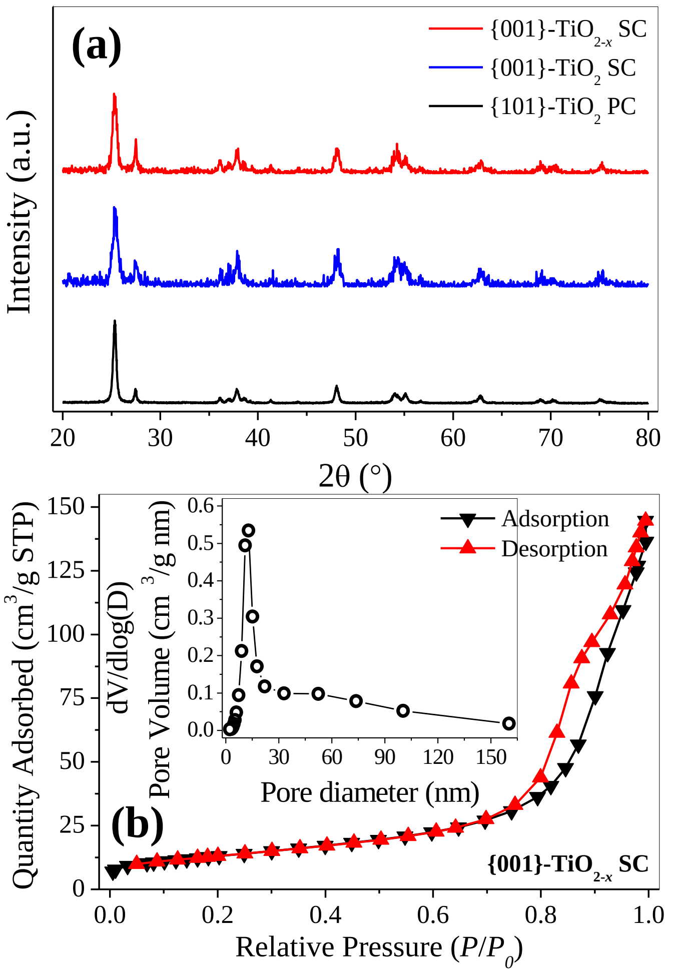

Supplementary Figure 2 | Crystal phase and surface area of different TiO_2 catalysts. XRD pattern (**a**) and BET spectra (**b**) of the {001}-TiO_{2-x} SCs, {001}-TiO_2 SCs and {101}-TiO_2 PCs.

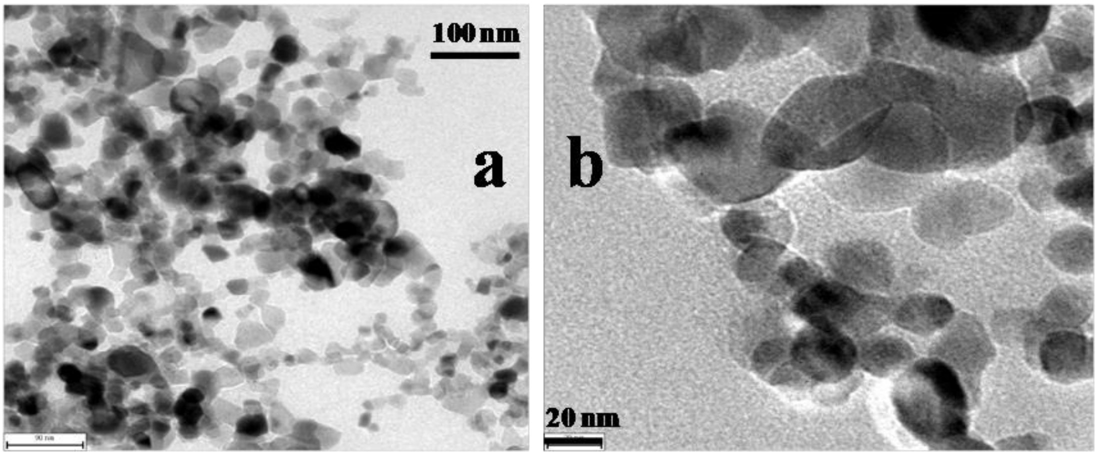

Supplementary Figure 3 | Morphological properties of commercial TiO_2. Typical TEM images of the {101}-TiO_2 PCs reference (**a**, **b**).

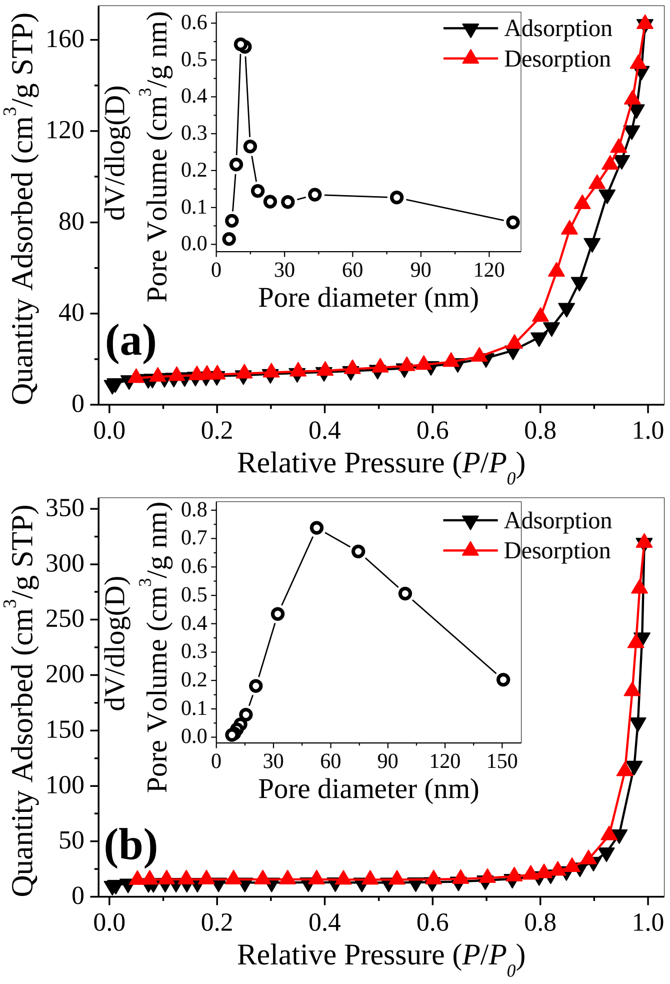

Supplementary Figure 4 | Surface area of two TiO_2 references. BET spectra of the {101}-TiO_2 PCs (**a**) and {001}-TiO_2 SCs (**b**).

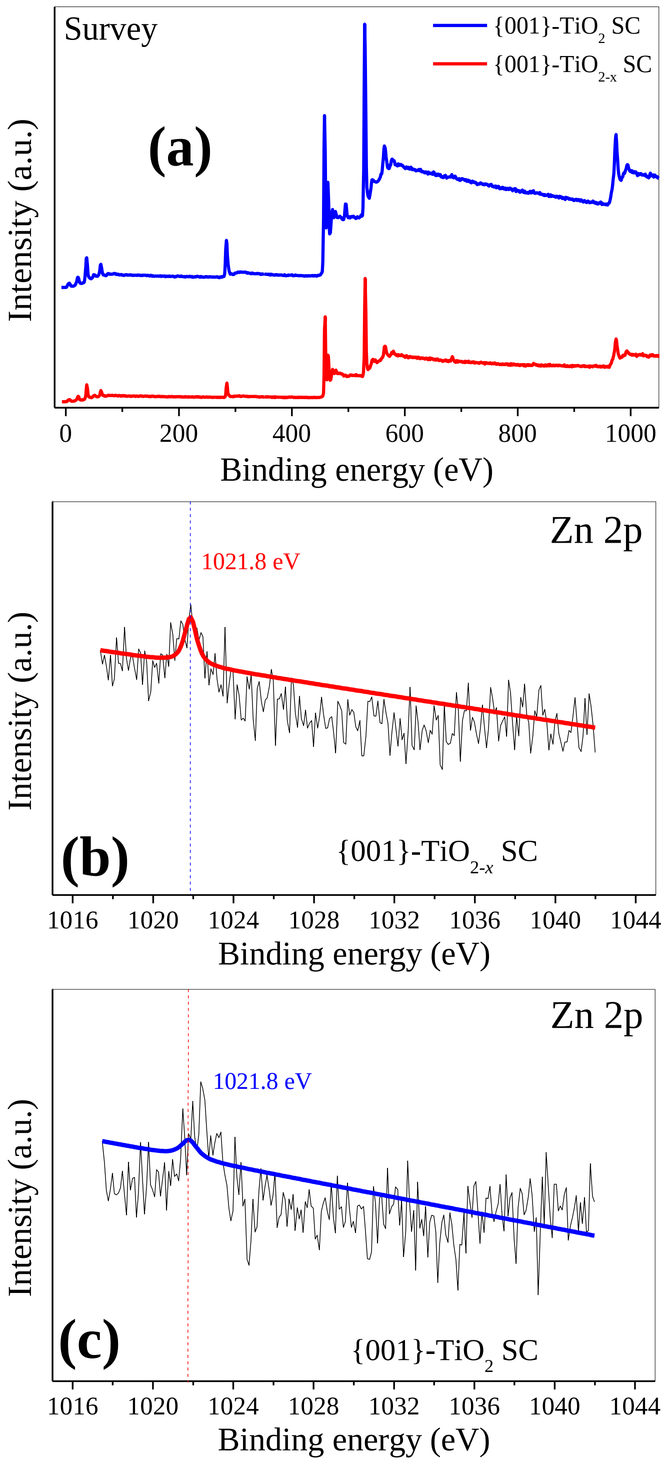

Supplementary Figure 5 | XPS spectra of different TiO_2 catalysts. XPS spectra of the survey and the Zn 2p of the {001}-TiO_{2-x} SCs (**a**, **b**) and the {001}-TiO_2 SCs (**a**, **c**).

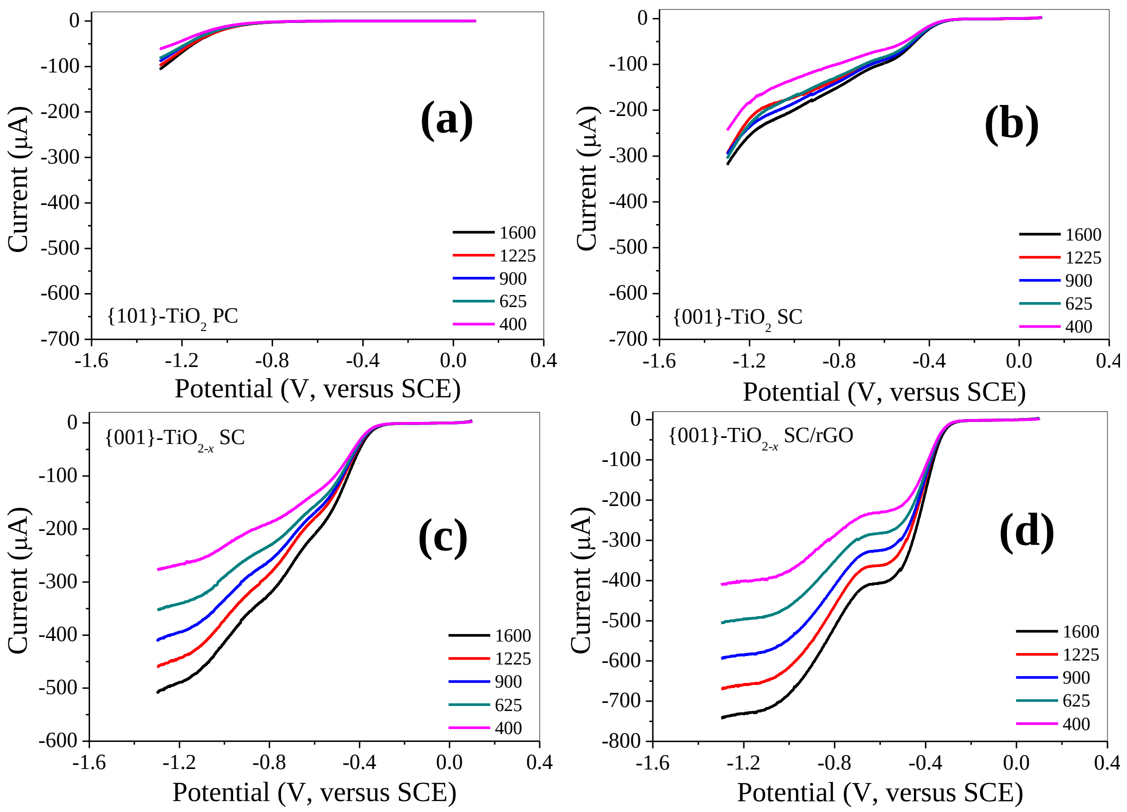

Supplementary Figure 6 | ORR behaviors of different TiO_2 catalysts at various rotating rates. Linear voltammetry in O_2-saturated 0.1 M aqueous KOH electrolyte solution at a scan rate of 10 mV s^{-1} at different RDE rotation rates on the {101}-TiO_2 PC (**a**), {001}-TiO_2 SC (**b**) and {001}-$TiO_{2\text{-}x}$ SC (**c**) and {001}-$TiO_{2\text{-}x}$ SC supported by home-made rGO (**d**).

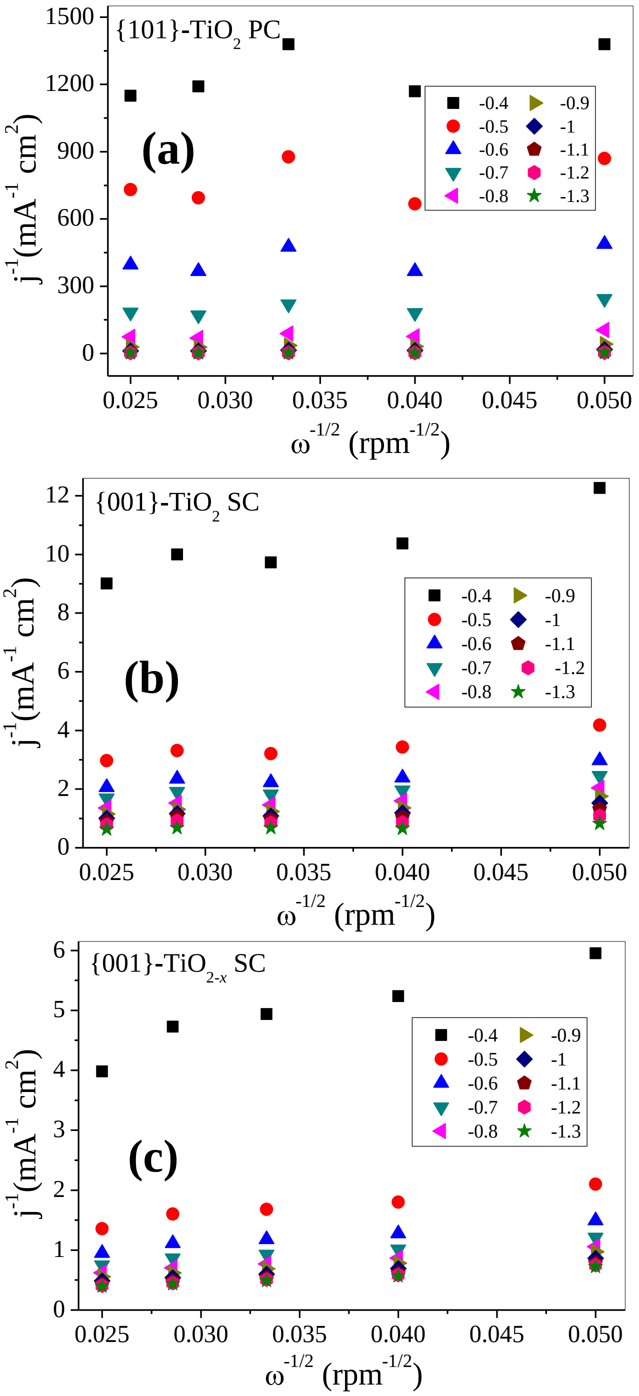

Supplementary Figure 7 | Calculation results from RDE tests. K-L plots (i^{-1} vs. $\omega^{-1/2}$) derived from the linear voltammetry in O_2-saturated 0.1 M aqueous KOH electrolyte solution at a scan rate of 10 mV s^{-1} at different RDE rotation rates on the {101}-TiO_2 PC (**a**), {001}-TiO_2 SC (**b**) and {001}-$TiO_{2\text{-}x}$ SC (**c**).

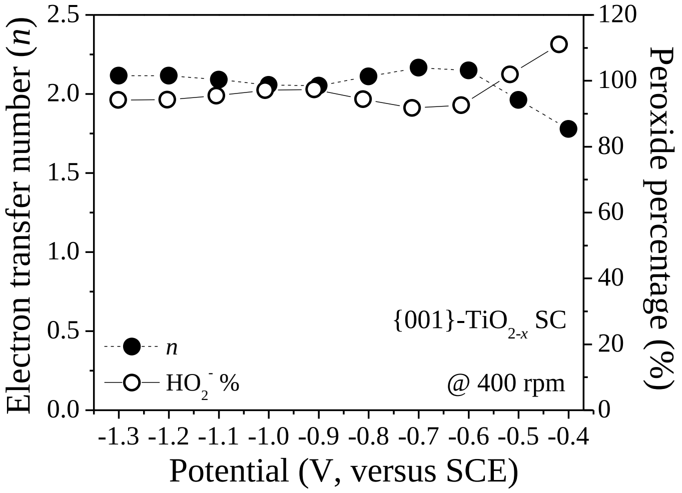

Supplementary Figure 8 | Calculation results from RRDE tests. Peroxide percentage and electron transfer number *n* of ORR on the defective {001}-TiO_{2-x} SCs in O_2-saturated 0.1 M aqueous KOH electrolyte solution at a scan rate of 10 mV s^{-1} with a rotation rate of 400 rpm.

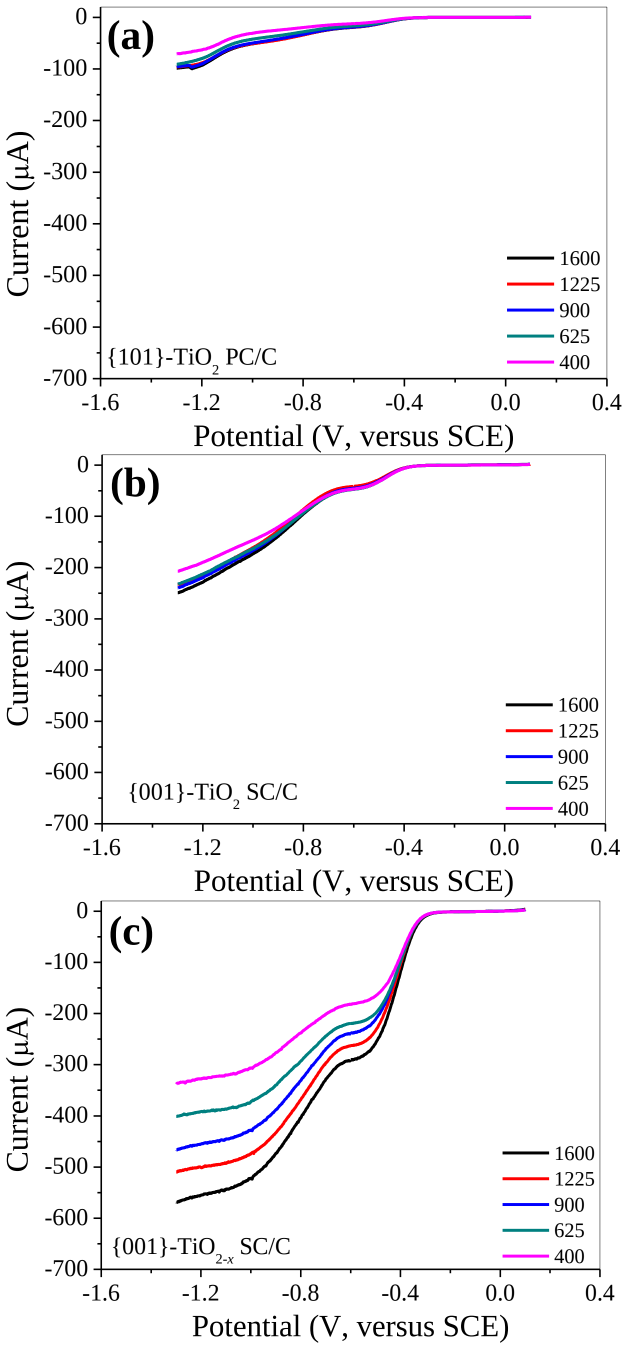

Supplementary Figure 9 | ORR behaviors of different TiO_2/C catalysts at various rotating rates. Linear voltammetry in O_2-saturated 0.1 M aqueous KOH electrolyte solution at a scan rate of 10 mV s^{-1} at different RDE rotation rates on the {101}-TiO_2 PC (**a**), {001}-TiO_2 SC (**b**) and {001}-TiO_{2-x} SC (**c**) supported by commercial carbon.

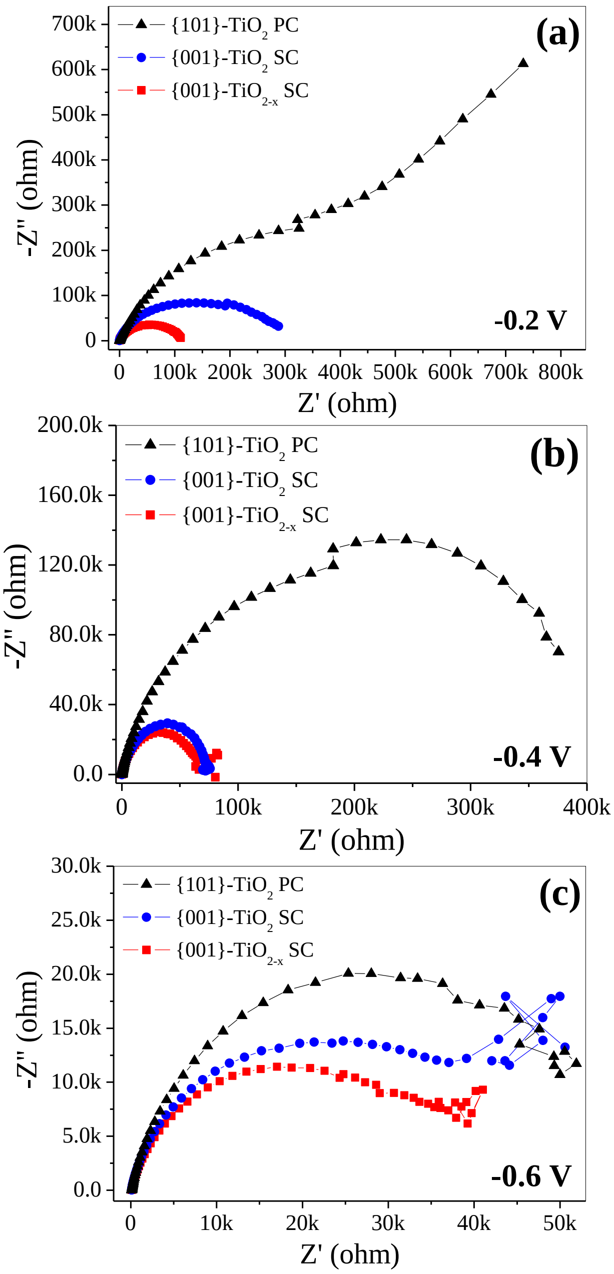

Supplementary Figure 10 | Electronic properties of different TiO_2 catalysts at various applied bias. EIS plots of the three anatase TiO_2 surfaces under the external bias of -0.2 V/SCE (**a**), -0.4 V/SCE (**b**) and -0.6 V/SCE (**c**).

Supplementary Table 1 Comparison of ORR specific activities of different TiO_2 catalysts in O_2-saturated 0.1 M aqueous KOH electrolyte solution (scan rate: 10 mV s^{-1}, rotation rate: 400 rpm).

ORR Catalyst	Specific Activity (×10^{-3} mA cm^{-2})		
	-0.35 V	-0.40 V	-0.45 V
{101}-TiO_2 PC	-1.50	-1.88	-2.41
{001}-TiO_2 SC	-12.11	-39.55	-106.65
{001}-TiO_{2-x} SC	-62.17	-180.06	-349.41
{101}-TiO_2 PC/C	-2.68	-9.43	-24.44
{001}-TiO_2 SC/C	-10.13	-34.89	-95.23
{001}-TiO_{2-x} SC/C	-180.82	-470.90	-748.66
{001}-TiO_{2-x} SC/rGO	-269.51	-622.19	-943.19

Supplementary Table 2 O-O bond length in the oxygen substrate and its key intermediates in ORR on {101}-TiO_2, {001}-TiO_2 and {001}-TiO_{2-x}.

Anatase TiO_2 surface	O_2 (Å)	O_2^{2-} (Å)	OOH^- (Å)
{001}-TiO_2	1.209	1.223	1.321
{001}-TiO_{2-x}	1.209	1.296	1.391
{101}-TiO_2	1.209	1.437	1.411

Supplementary Table 3 Calculated thermodynamic properties (ΔG) and energy barrier (E_a) in ORR on {001}-TiO_2, {001}-TiO_{2-x} and {101}-TiO_2, at 298.15 K and 1 atm.

ORR Step	{001}-TiO_2		{001}-TiO_{2-x}		{101}-TiO_2	
	ΔG	E_a	ΔG	E_a	ΔG	E_a
O_2* + 2e$^-$ → O_2^{2-}*	-2.478	–	-3.296		3.945	
O_2^{2-}* + H_2O → OOH^-* + OH^-	-0.833	-0.091	-0.923	2.889	-3.400	2.950
OOH^-* → O* + OH^-	2.648	2.900	1.372	2.030	-3.400	0.800
O* + H_2O + e$^-$ → OH* + OH^-	-2.806	7.550	-4.431	5.007	-2.583	10.582
OH* + e$^-$ → OH^-	-0.139	0.430	-0.463	-0.302	1.887	-3.080
Total	-3.608		-7.741		-3.551	

Supplementary Note 1: ORR Properties on the Different Surfaces of Anatase TiO_2. To date, the O_2 adsorption configurations on {101}-TiO_2 and {001}-TiO_2 have been studied extensively. It is generally agreed that {001}-TiO_2 is relatively more active for O_2 adsorption than {101}-TiO_2, and also the 5-fold coordinated Ti^{4+} surface ions are the active site for O_2 adsorption site[1]. In this study, to accurately understand the possible contribution of oxygen vacancy to ORR, we first modeled the O_2 adsorption onto a 5-fold coordinated Ti^{4+} surface ion with two Ti-O bonds on {001}-TiO_2 and {001}-$TiO_{2\text{-}x}$ for comparison (Table 1). For {001}-TiO_2, O_2 was weakly bound, with a low ΔE_{ads} of 1.030 eV, compared to that onto {001}-$TiO_{2\text{-}x}$ with a much higher ΔE_{ads} of 2.277 eV. Solid-liquid interfacial adsorption is usually the essential first step in heterogeneous catalysis[2]. This result indicates that the O_2 adsorption capacity onto {001}-TiO_2 could be significantly enhanced by crystal oxygen vacancy.

It is widely agreed that adsorbed O_2 is initially reduced to O_2^- [3], which is the precursor of other further reduced active species, such as O_2^{2-} and HOO^-. Moreover, on metal oxide catalyst surface, the ORR in alkaline media is generally proposed to proceed through the chained steps including peroxide and oxide formation, hydroxide displacement, and hydroxide regeneration[4-6]. Thus, the ORR mechanism on anatase TiO_2 surface could be described in Fig. 7a, similar to the series two-electron pathway of ORR on metal catalysts[7], and the key intermediates of O_2^-, O_2^{2-}, HOO^- and OH^- were detected by in situ infrared measurements[8].

The key intermediates of ORR on anatase TiO_2 surfaces involve the active

species of O_2^{-}*, O_2^{2-}*, HOO^{-}*, OH* and O* (*: adsorbed state). Their geometry structures of adsorption configuration on {001}-TiO_2, {001}-TiO_{2-x} and {101}-TiO_2 were thermodynamically optimized, as illustrated in Fig. 6, to understand their catalytic activities. As anticipated, all the O-O bonds in O_2^{-}*, O_2^{2-}*, HOO^{-}* were elongated over that of O_2 (Supplementary Table 2), indicating their thermodynamic activation for further ORR. The ORR could be divided into two stages (Fig. 7a): reducing absorbed O_2 to adsorbed peroxide and peroxide decomposition, followed by the regeneration of hydroxide from absorbed oxide.

Supplementary References

1. Mattioli, G.; Filippone, F.; Bonapasta, A. A., Reaction intermediates in the photoreduction of oxygen molecules at the (101) TiO_2 (anatase) surface. *J. Am. Chem. Soc.* **128**, 13772-13780 (2006).
2. George, C.; Ammann, M.; D'Anna, B.; Donaldson, D. J.; Nizkorodov, S. A., Heterogeneous photochemistry in the atmosphere. *Chem. Rev.* (In the press) DOI: 10.1021/cr500648z (2015).
3. Wang, Z. H.; Ma, W. H.; Chen, C. C.; Ji, H. W.; Zhao, J. C., Probing paramagnetic species in titania-based heterogeneous photocatalysis by electron spin resonance (ESR) spectroscopy-A mini review. *Chem. Eng. J.* **170**, 353-362 (2011).
4. Suntivich, J.; Gasteiger, H. A.; Yabuuchi, N.; Nakanishi, H.; Goodenough, J. B.;

Shao-Horn, Y., Design principles for oxygen-reduction activity on perovskite oxide catalysts for fuel cells and metal-air batteries. *Nat. Chem.* **3**, 647-647 (2011).

5. Cheng, F. Y.; Chen, J., Metal-air batteries: from oxygen reduction electrochemistry to cathode catalysts. *Chem. Soc. Rev.* **41**, 2172-2192 (2012).
6. Cheng, F. Y.; Zhang, T. R.; Zhang, Y.; Du, J.; Han, X. P.; Chen, J., Enhancing electrocatalytic oxygen reduction on MnO_2 with vacancies. *Angew. Chem. Int. Edit.* **52**, 2474-2477 (2013).
7. Farberow, C. A.; Godinez-Garcia, A.; Peng, G. W.; Perez-Robles, J. F.; Solorza-Feria, O.; Mavrikakis, M., Mechanistic studies of oxygen reduction by hydrogen on PdAg(110). *ACS Catal.* **3**, 1622-1632 (2013).
8. Nakamura, R.; Imanishi, A.; Murakoshi, K.; Nakato, Y., In situ FTIR studies of primary intermediates of photocatalytic reactions on nanocrystalline TiO_2 films in contact with aqueous solutions. *J. Am. Chem. Soc.* **125**, 7443-7450 (2003)
